# Rapid assessment of viable but non-culturable *Bacillus coagulans* MTCC 5856 in commercial formulations using Flow cytometry

**DOI:** 10.1371/journal.pone.0192836

**Published:** 2018-02-23

**Authors:** Muhammed Majeed, Shaheen Majeed, Kalyanam Nagabhushanam, Ardra Punnapuzha, Sheena Philip, Lakshmi Mundkur

**Affiliations:** 1 Sami Labs Limited, Peenya Industrial Area, Bangalore, Karnataka, India; 2 Sabinsa Corporation, Payson, UT, United States of America; 3 Sabinsa Corporation, East Windsor, NJ, United States of America; 4 Biological Research Department, Sami Labs Limited, Bangalore, Karnataka, India; Massachusetts Institute of Technology, UNITED STATES

## Abstract

Accurate enumeration of bacterial count in probiotic formulation is imperative to ensure that the product adheres to regulatory standards and citation in consumer product label. Standard methods like plate count, can enumerate only replicating bacterial population under selected culture conditions. Viable but non culturable bacteria (VBNC) retain characteristics of living cells and can regain cultivability by a process known as resuscitation. This is a protective mechanism adapted by bacteria to evade stressful environmental conditions. *B*. *coagulans* MTCC 5856(LactoSpore®) is a probiotic endospore which can survive for decades in hostile environments without dividing. In the present study, we explored the use of flow cytometry to enumerate the viable count of *B*. *coagulans* MTCC 5856 under acidic and alkaline conditions, high temperature and in commercial formulations like compressed tablets and capsules. Flow cytometry (FCM) was comparable to plate count method when the spores were counted at physiological conditions. We show that VBNC state is induced in *B*. *coagulans* MTCC 5856by high temperature and acidic pH. The cells get resuscitated under physiological conditions and FCM was sensitive to detect the VBNC spores. Flow cytometry showed excellent ability to assess the viable spore count in commercial probiotic formulations of *B*. *coagulans* MTCC 5856. The results establish Flow cytometry as a reliable method to count viable bacteria in commercial probiotic preparations. Sporulation as well as existence as VBNC could contribute to the extreme stability of *B*. *coagulans* MTCC 5856.

## Introduction

Probiotics are defined as “live microorganisms, which when administered in adequate amounts, confer a health benefit on the host” as accepted by the Food and Agriculture Organization of the United Nations/World Health Organization and the International Scientific Association for Probiotics and Prebiotics [1, 2]. Probiotic supplements have shown exponential growth in the last decade and are now available in different formulations, and as probiotic enriched food and beverages[3].These organisms are presumed to mediate the beneficial effects by various mechanisms, including immune modulation, anti microbial activity against gut pathogens, alleviating lactose intolerance, hypercholesterolemia and other inflammatory diseases of the gut [4, 5]. Viability is considered as an important aspect for the function of probiotics, hence it is critical to accurately enumerate the population of viable microbes in any formulation.[6]

During the process of formulation, probiotic bacteria may enter a dormant state, where they are metabolically active but are not culturable[7]. Intermediate states between viable and dead bacteria like injured and stressed cells are difficult to detect by the plating method and are often termed as viable but nonculturable state (VBNC),a protective response by the bacteria to evade the stressful condition [7, 8]. Extensive molecular studies have confirmed that VBNC is a distinct viable state of bacteria [9, 10]. This is a strategy employed by bacterial species to enter a state of very low metabolic activity, allowing them to survive under unfavourable conditions, but prevents them from forming colonies[11]. VBNC microbes can regain their ability to grow, once they encounter a suitable environment which is also known as known as resuscitation [12, 13]. VBNC is most suitable for non- sporulating bacteria as it offers a greater advantage for them to survive the hostile environment. Since standard plating techniques estimate only the replicating bacteria, they may not accurately enumerate the VBNC in the given probiotic preparation. Several alternative methods, such as fluorescent in situ hybridization (FISH), polymerase chain reactions, microplate fluorochrome assay, propidium monoazide (PMA) real-time quantitative polymerase chain reaction and flow cytometry offer the potential to enumerate both culturable and VBNC bacteria [14–22].

In the last two decades Flow cytometry (FCM) has been used widely as a tool to investigate bacteria in laboratory cultures, environmental, clinical and food samples [23]. It is a sensitive technique to determine the cell number and their heterogeneity at rates of 100 to 1,000 cells per second[24]. Various fluorescent probes are used for the assessment of bacterial viability[25].

Fluorescent probes applied in the viability assessment of bacteria include DNA binding dye exclusion probes like propidium iodide (PI), which are impermeable in cells with intact membrane and staining indicates dead cells [26, 27]. Green fluorescing SYTO BC™ stains are high-affinity nucleic acid stains that easily penetrate all cells and are used for assessing total cell counts, and physiological indicators like fluorescein diacetate, bis-(1,3-dibutylbarbituric acid) trimethineoxonol (DiBAC_4_)and Calcein AM which fluoresce only in live cells. Calibrated suspension of polystyrene microspheres is included along with SYTO BC™ to count the bacterial cell numbers in FCM [16, 28–30].

*B*. *coagulans* MTCC 5856 is a probiotic strain marketed under the trade name LactoSpore® for the past two decades. It is a non GMO probiotic with GRAS status, with an ability to withstand high temperature and shows genetic stability through several years of commercial production [31–33]. This bacterium can form endospores and can survive for decades in hostile environments without dividing. The stability of the bacteria to unfavourable conditions was thought to be only due to sporulation and the existence of a VBNC state has not been explored so far.

Although FCM has been used for the enumeration of different vegetative bacterial probiotics, the spores of *B coagulans* have not been subjected to FCM analysis. Further, commercial production and formulation can induce different physiological states in the probiotic culture. In the present study we describe the value and benefits of FCM as a method to detect and enumerate live spores, vegetative cells and VBNC of *B coagulans* MTCC 5856 (LactoSpore®).

## Methods

### Test material

*Bacillus coagulans* MTCC 5856 samples were manufactured by Sami Labs Limited (Bangalore, India) following a proprietary, in-house, good manufacturing practice. Pure *B*. *coagulans* MTCC 5856 spores were spray–dried and standardised with food grade maltodextrin (Sanwa Starch Co.Ltd. Kashihara, Nara, Japan) to achieve a desired concentration of 15 X 10^9^ CFU per gram of the finished product in powder form. The tablets and capsules were manufactured by Sabinsa Corporation, Payson, UT, USA. Commercial preparations of other probiotic formulations A—C were purchased from the US market, while probiotic D was purchased from a departmental store in India. All the commercial products were stored as per the manufacturer’s instructions and used before their expiry date. Probiotic E was a commercial tablet of *B*. *coagulans* MTCC 5856.

### Sample preparation for flow cytometry

For FCM analysis and viable count estimation, 10 mg of spores (15X10^9^/g) were suspended in 1 ml of sterile phosphate buffered saline (PBS, pH 7.4)and incubated in a water bath for 30 min at 75°C, followed by immediate cooling to below 45°C. This suspension was taken for either FCM analysis or further serially diluted in sterile PBS and the viable count was enumerated by plating on glucose yeast extract agar (HiMedia, Mumbai, India) as described earlier[33].One set of spores were analysed by FCM without the activation step.Each experiment was repeated thrice in duplicates. Average mean of spore viable counts were expressed in log_10_ CFU.

### Staining

The fluorescent stains used were cFDA, propidium iodide (PI), and SYTO BC™ and calibrated suspension of polystyrene microspheres (Thermo Scientific Inc.). Double staining with cFDA and PI were carried out by incubating the spore suspension (10^7^/ml) with 50μM cFDA at 37°C for 60 minutes in and 10μM PI in the last 10 min. The time for cFDA staining was standardized earlier at different times and 60 minutes was found to be optimum. SYTO BC™ was diluted to 1000 times and the microbeads 100 times to get 1X concentration of SYTO BC™ and 1 X10^6^ beads/ml. Spores were stained at37°C for 10 min in a separate tube.

### Flow cytometry

FCM acquisition was performed with a Canto II cytometer, and the data analysis with Diva software 6.2.1(BD biosciences, CA, USA). Cytometry set up and tracking (CST) beads (BD biosciences) were used to standardize the flow cytometer setup as per the manufacturer’s instructions. All the buffers used for flow cytometry were filtered through 0.2 micron filter to prevent background bacterial noise. The filtered buffers were run as a negative control and threshold values were set at 2000. Forward and side scatter voltages for the photomultiplier tube (PMT) were adjusted by running the sterile buffer and buffer containing bacteria. Fluorescent voltages were adjusted based on stained and unstained samples. Compensation controls were run with each set of experiments to minimise the overlap of FITC (cFDA) & PE (PI) during double staining. The final PMT voltage parameters were set at FSC-430 units, SSC-425 units, FITC-280 units and PE-269 units. Auto florescence gating was carried out using unstained spores.Calibrated suspension of polystyrene microspheres can be distinguished from SYTO BC™ on a plot of forward scatter versus fluorescence. The density of the bacteria in the sample can be determined from the ratio of bacterial signals to microsphere signals in the cytogram.

### Calculations

Total number of spores as detected by SYTO BC™ staining was calculated by

Total No of spores # Events inSYTO BC™ ^+^ region

                = —————————————X 10^6^ X dilution factor (DF)

                # Events in beads

The numbers of viable spores were calculated by

No of viable spores # Events in CFDA^+^ region

                = ———————————- X 10^6^ X dilution factor (DF)

                # Events in beads

Or

No of viable spores Total spores—# Events in PI^+^ region

                = ——————————————X 10^6^ X dilution factor (DF)

                # Events in beads

### Validation of flow cytometry

A spore stock of 15X 10^7^ spores /ml was prepared from *B*. *coagulans* MTCC 5856 (strength of 15X10^9^/g). The spore suspension was serially diluted in PBS and the count was enumerated from each dilution individually by FCM and compared with plate count. To understand the differentiation between spores and vegetative cells, spores were grown in nutrient broth (HiMedia, India) overnight and the cells were either analysed individually or after mixing with fresh spores. Alternately, the vegetative cells were exposed to isopropyl alcohol and then mixed with spores to show the sensitivity of FCM in counting live and dead cells.

### VBNC and resuscitation

To study the proportion of VBNC spores and their resuscitation under favourable environment for germination, the spore suspension (15X10^9^/ml) was incubated in 0.1M acetate buffer (pH 2) for 24 hours. To induce temperature stress, the spore suspension (15 X10^9^/ml) was incubated at 140°C for 5 min. The spores were centrifuged, washed with PBS twice and suspended in nutrient broth and incubated at 37°C for 60 minutes. Viable spores were enumerated by both FCM and plate count.

### Spore count in commercial preparations

The entire content of the capsule was suspended in 50 ml PBS while the tablet was weighed and crushed and thoroughly and suspended in 50ml of PBS. The samples were analysed by FCM as well as plate count as described earlier.

### Spore count in orange juice

Predetermined number spores with overages were mixed in commercial orange juice purchased from the market. The juice was stored at 4°C for 24 hours and the number of spores were determined by both FCM and plate count method.

### Commercial probiotics

Four probiotics were taken for comparison with *B*. *coagulans* MTCC 5856. They were purchased from local supermarkets or obtained from the manufacturers or distributors. All were stored according to label and all were used for study before their expiry dates. The commercial tablets/capsules were crushed and incubated for 24 hours in 0.1M acetate buffer (pH 2), phosphate buffer (pH 7.2) or Tris buffer (pH 9.2). The tablets and capsules were processed for flow cytometry without the activation step as described earlier. The experiment was repeated thrice in duplicates.

### Statistical analysis

Graph Pad prism software version 5.01(GraphPad Software, Inc., La Jolla, CA, USA). Results were expressed as Mean± Standard deviation Mann-Whitney test was used for analysis of significance and a P value of <0.05 was considered statistically significant.

## Results

### Enumeration of *B coagulans* Spores by flow cytometry

**Fig 1:**
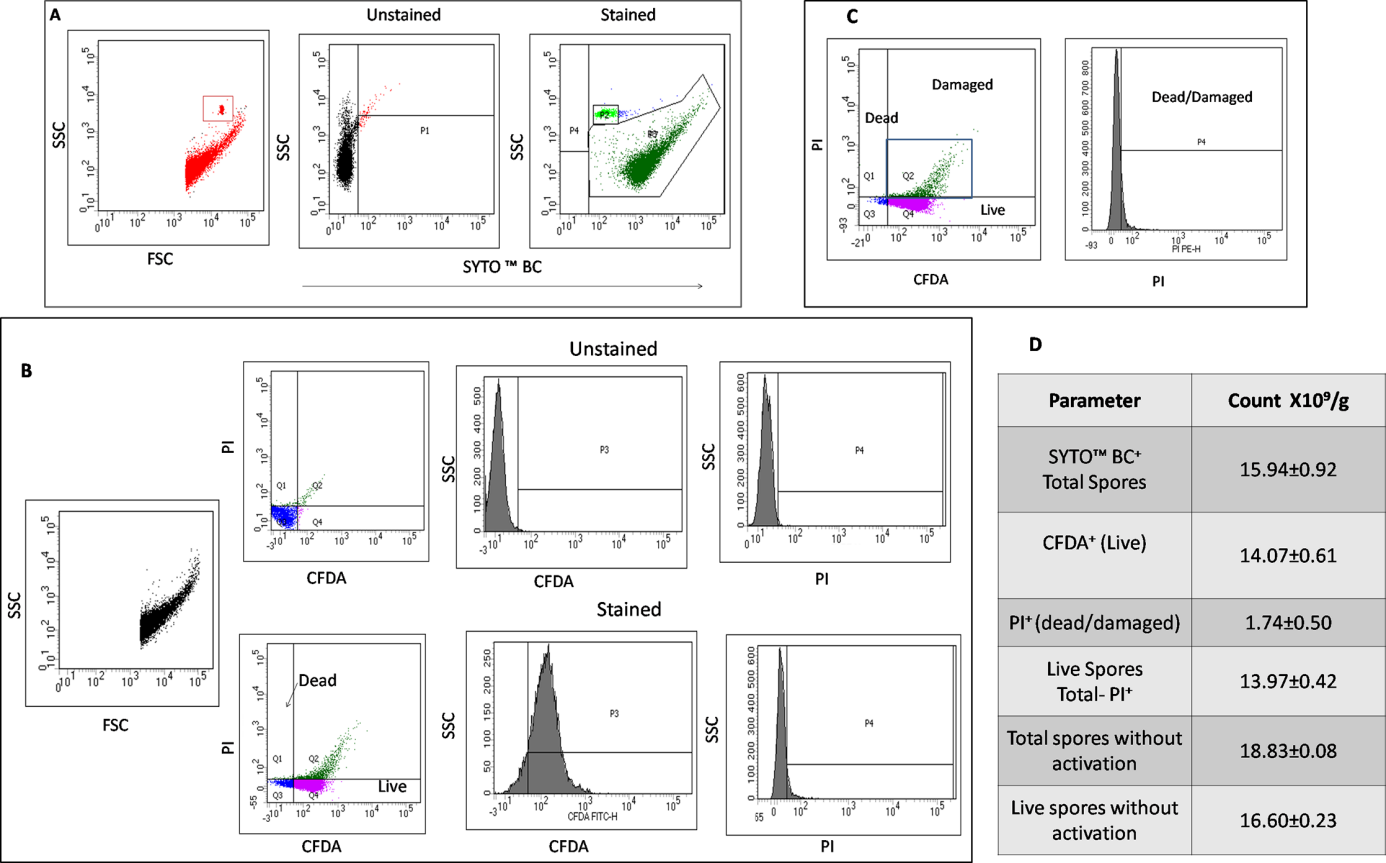
Flow cytometric analysis of B coagulans MTCC 5856 spores. 10 mg of dry spores were suspended in 1ml of PBS and activated for 30 min at 75°C, followed by fluorescent staining as described in the methods section. Data represents mean± standard deviation of at least 3 independent experiments. A: Gating strategy for SYTOBC ™ staining- The microbeads and the stained spores are gated separately based on unstained control B: Gating strategy for CFDA and PI staining. The dot plot represents a double staining for both CFDA and PI. The quadrant 4(Q4) represents live cells, Q1- dead cells and Q2 damaged cells. The double positive cells were not considered for enumerating the live spores. C: Representative dot plot for the FCM analysis of Live and Dead spores D: Table showing the calculation of viable spores.

The spores were distributed in four quadrants of the dot plot following Flow cytometry. The quadrant 1(Q1) represented the PI stained dead cells; Q2 had spores which were stained by both PI and CFDA, which were categorised as damaged cells. Live spores could be seen as CFDA positive cells in Q3, while few spores were seen in Q4 which were unstained spores. We considered the CFDA ^+^PI^-^(Q3) cells as live spores for the enumeration of spore counts. Total count of spores as determined by SYTO BC™ staining was found to be 15.72±0.92X10^9^/g. The viable spores as calculated by (CFDA+) cells was found to be 14.07±0.62X10^9^/gram and that by subtracting the PI positive (dead) cells from total count was 13.97±0.42 X10^9^/g(Fig 1), which were highly comparable. The total numbers as well as live count were slightly higher when the activation step was omitted before FCM analysis (16.6±0.23).(Fig 1).

### Validation of flow cytometry

**Fig 2:**
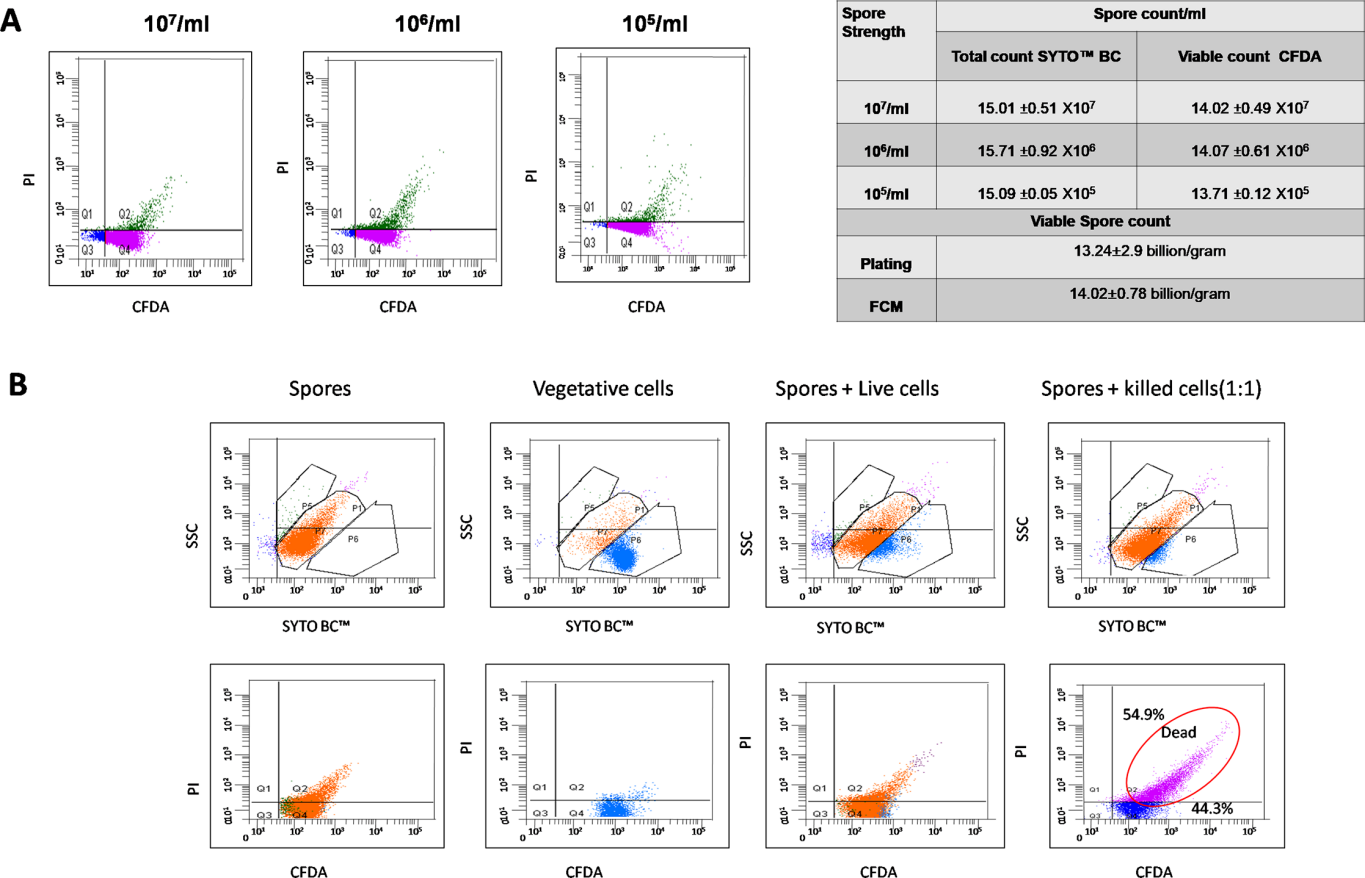
Validation of flow cytometry. A: Spore suspension was diluted and each dilution was analysed by FCM as described earlier. Quantitative analysis is given in the table. B: FCM analysis of spores, vegetative cells and a mixture of spores and vegetative cells and killed cells.

To validate the method of spore enumeration by FCM, we diluted the spore stock and counted the total number and viable spores by FCM in each dilution. As shown in Fig 2, FCM was sensitive to count the spores from a stock of 10^7^ to 10^5^per ml.Tenfold dilution of spores was reflected in the FCM count resulting in comparable total and viable count per gram of spores irrespective of the dilution used for enumeration. Dilution below 10^4^ spores per ml, were not taken as it was not feasible to collect 10000 events for the FCM analysis. The viable spore count of freshly prepared suspension of spores by FCM (14.02±0.78 billion/g) was highly comparable to the plate count (13.24±2.9billion/g), further validating the method for the enumeration of *B*. *coagulans* MTCC 5856. To understand the difference between spores and vegetative cells, we allowed the spores to germinate and grow in nutrient broth overnight, and analysed the cells by FCM. The vegetative cells were seen as a distinct population in comparison to spores in the SYTO BC™ stained plots. The live and dead staining by CFDA and PI could not differentiate the spores from vegetative cells. To establish the sensitivity of the assay, we used killed vegetative cells mixed with spores for FCM analysis in equal proportion (Fig 2). Mixing spores and killed cells showed an increase in dead cells as stained by PI (54.9%) while live spores were 44.3%. These results establish the robustness of FCM as an accurate technique to identify spores, vegetative cells and the number of viable and dead cells in the given population (Fig 2).

### VBNC and resuscitation

**Fig 3:**
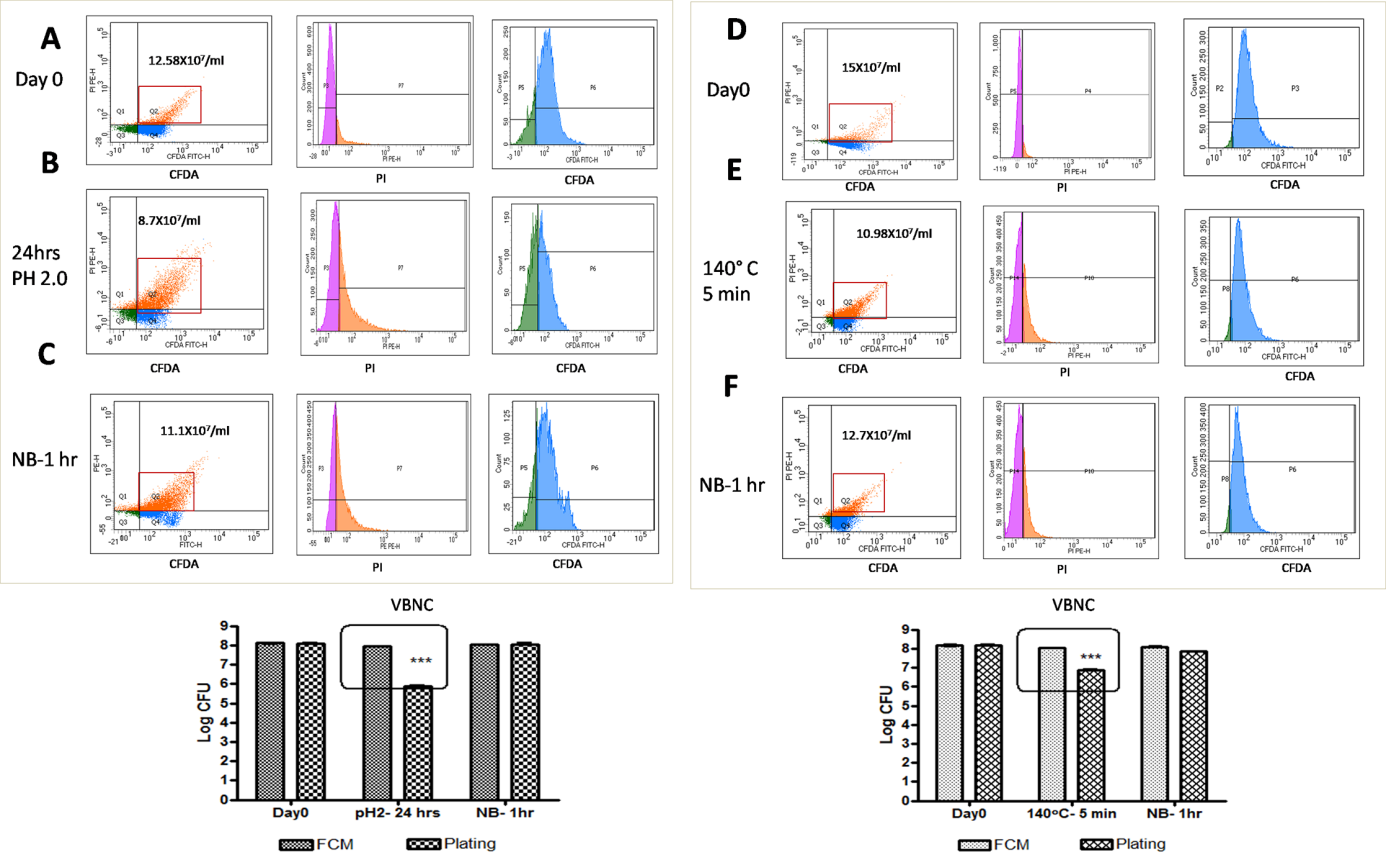
VBNC and resuscitation. The spore suspension (15 X10^9^/ml) was incubated in 0.1M acetate buffer (pH 2) for 24 hours, or incubated at 140°C for 5 minutes followed by washing and suspension in Nutrient Broth (NB) and incubated at 37°C for 60 minutes. The viable spores were enumerated by both FCM and plate count as described earlier. Data represents mean± standard deviation of at least 3 independent experiments.

VBNC is a state which is induced by unfavourable environmental conditions. We used acidic pH and high temperature to induce VBNC in *B*. *coagulans* MTCC 5856. The live count of spores before incubation in acidic buffer was 13.12±0.76 X10^7^ /ml by FCM and 12.5±0.84X10^7^ /ml by plating. After 24 hours of incubation at room temperature in acetate buffer at pH 2.0, the, spore count estimated by plating reduced to 7.25±1.06X10^5^ /ml, while it was 8.7±0.19X10^7^ /ml by FCM. Interestingly the numbers of spores in the double positive quadrant were higher after 24 hours exposure to stressful condition, suggesting cell damage. Incubation in nutrient broth for 60 minutes restored the spore viability as seen by an increase in plate count to 11.5±2.1X10^7^ /ml and 11.13±0.31X10^7^ /ml by FCM (Fig 3A–3C). Similar effect was observed by heat treatment at 140°C for 5 minutes. Increase in temperature resulted in 1.5 log reduction in plate count (14.7±1.7x10^7^to7.7±0.7x10^6^/ml) which was restored back to 6.9±0.7x10^7^on incubation in nutrient broth. FCM was found to enumerate the VBNC as the count remained at 10.9±0.8x10^7^and 12.3±.6 x10^7^ following heat treatment and nutrient broth resuscitation (Fig 3D–3F).

### Spore count in commercial preparations

**Fig 4:**
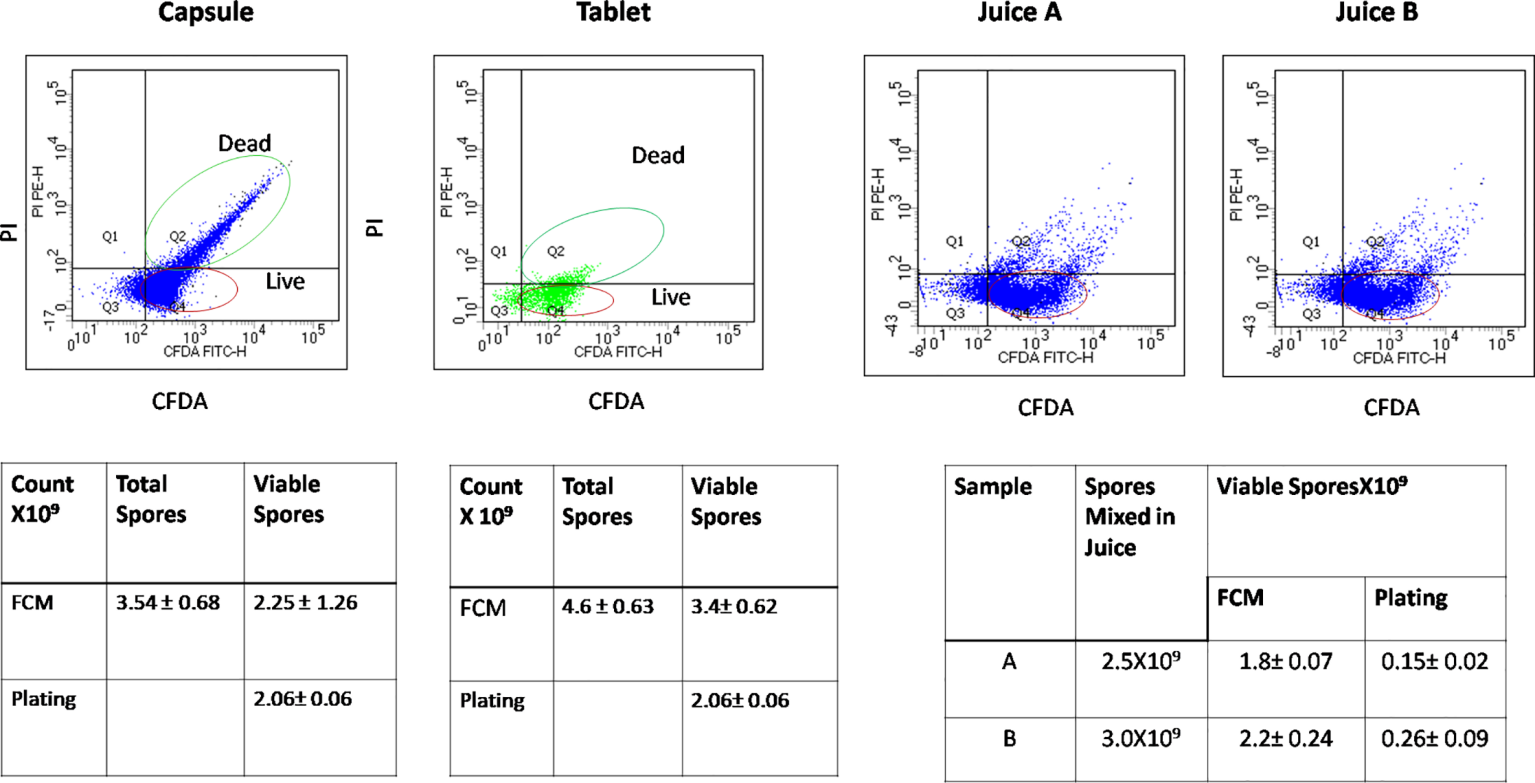
Spore viability in commercial preparations. Viability of B coagulans spores in tablets (A) capsules(B) and Orange juice(C): Total count and live spore count were determined by FCM and plating method. Data represents mean± standard deviation of 3 independent experiments.

We then enumerated the viability of spores in capsules and tablet formulation since the process involved in the manufacture is known to affect the viability of probiotics. The numbers of viable spores were observed to be 2 billion in both capsule and tablet which was in accordance with the label on the capsules. The total count by FCM was found to be 3.54±0.68 billion / capsule while it was 4.6±0.63billion/g in the tablet. The viable spores in the tablet as enumerated by FCM were 3.4±0.62 X10^9^ while in the capsule it was 2.25±0.26 X10^9^, while the plate counts were2.1±0.14 X10^9^ and 2.06±0.06 X10^9^ in tablets and capsules respectively. The numbers of viable spores were not less than 2 billion as per the label in the commercial product by both FCM analysis and plate count (Fig 4A and 4B)

### Spore viability in orange juice

We next studied its viability in orange juice, which is a direct application of the probiotic in the market. Spores were added to commercial orange juice and the viability estimated by both FCM and plate count method after 24 hours. As shown in Fig 4C, the spore count by FCM was close to the number of spores added to the juice, while plating method showed one log lower count. This result suggests that FCM method was adaptable for the enumeration of spores from commercial preparation such as orange juice with considerable accuracy.

### Comparative stability of probiotics

**Fig 5:**
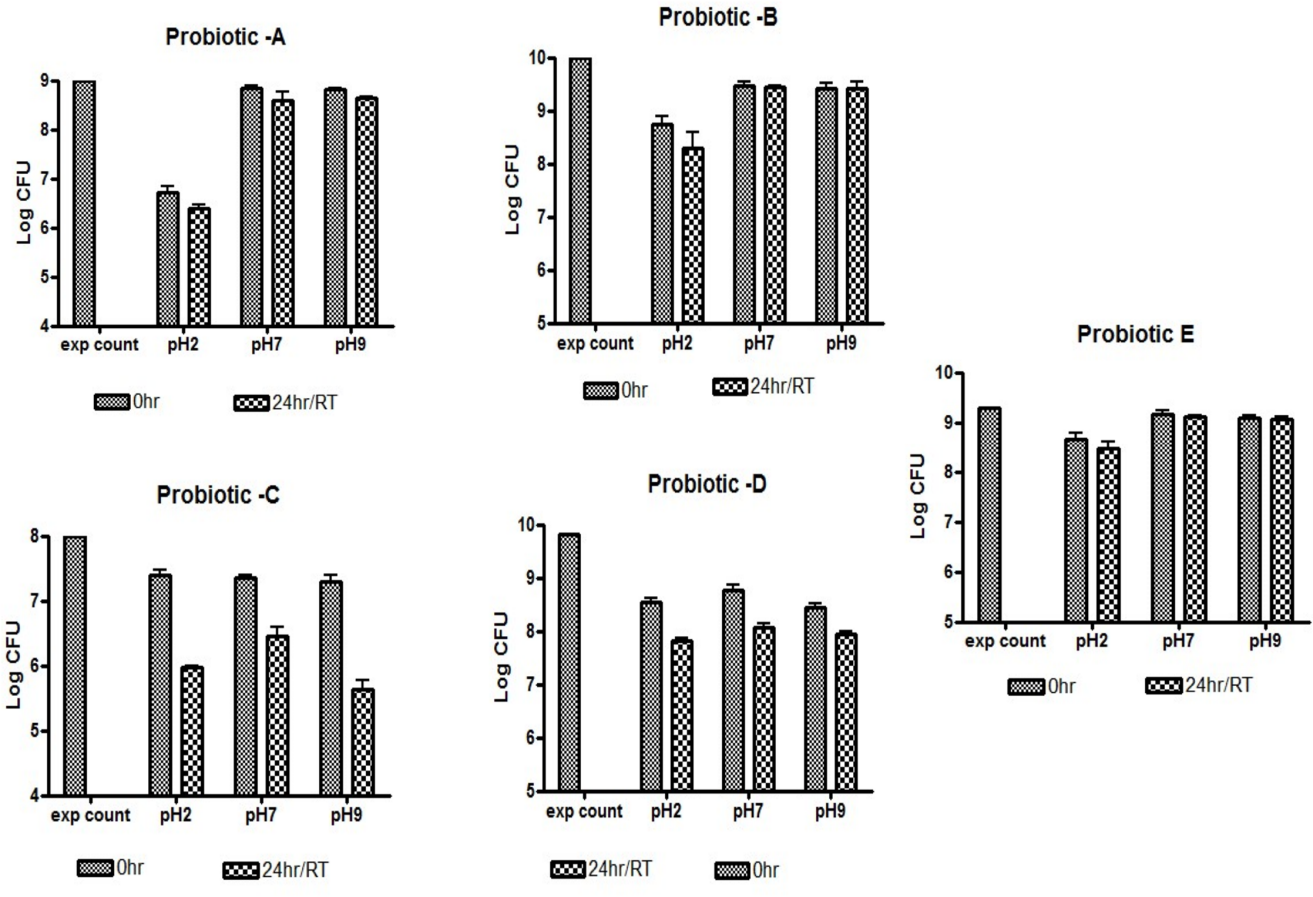
Comparative stability of probiotics. Bacterial count was assessed by flow cytometry as described earlier. The probiotic formulations A—C were purchased from the US market, while probiotic D was purchased from India. All the commercial products were stored as per the manufacturer’s instructions and used before their expiry date. Probiotic A: Commercial tablet of *Bifidobacterium infantis*, Probiotic B:Commercial tablet of *Lactobacillus rhamnosus* GG, Probiotic C:Commercial tablet of *Lactobacillus acidophilus*, Probiotic D:Commercial preparation of *Lactobacillus casei* and Probiotic E:Commercial tablet of *B coagulans* MTCC 5856. CFDA positive cells were taken as live cells. Data represents mean± standard deviation of 3 independent experiments.

Stability is an important criterion for probiotics. Therefore we compared the viable count of commercial probiotic formulations with *B*. *coagulans* MTCC 5856 at different conditions. B coagulans MTCC 5856 (Probiotic E) showed viability at neutral and alkaline pH and less than one log reduction in CFU at acidic condition even after 24 hours of incubation at room temperature. Probiotic A (*Bifidobacterium infantis*) was stable at neutral and alkaline pH, but a reduction of 2.5 to 3 log in CFU was observed at acidic conditions. The CFU of Probiotic B (*Lactobacillus rhamnosus* GG) was a 1.5 log lower than expected value at pH2, while neutral and alkaline conditions did not affect the total count. By 24 hours 2 log reduction in CFU was observed at acidic pH. Probiotic C (*Lactobacillus acidophilus*) showed less than a log reduction under all three conditions initially, while 1 and 2 log reduction in CFU was observed after 24 hours at neutral and acidic/ alkaline conditions respectively. Probiotic D (*Lactobacillus casei*) showed comparable stability at all three conditions but 2–3 log reduction in CFU was observed after 24 hours incubation at RT (Fig 5). Commercial Probiotics C and D were found to rapidly lose viability at room temperature but showed better stability when stored at 4°C.

## Discussion

In the present study we show that Flow Cytometry is a sensitive and effective method to enumerate the *B coagulans* MTCC 5856 spores, under normal and stressed conditions. FCM over estimated the viable counts in comparison to plate counts which could be related to the non culturable cells present in any preparation of probiotic, which could not be enumerated by the standard method. Exposure of stressed cells to growth media could resuscitate and restore the plate count, suggesting that FCM is an accurate method to count viable bacterial spores in commercial preparations.

*B coagulans* MTCC 5856 an anaerobic, spore forming, thermo tolerant bacterium with a long history of being used as a probiotic [31, 33]. Apart from being a safe dietary supplement, *B*. *coagulans* MTCC 5856 has shown therapeutic benefits in the treatment of irritable bowel syndrome [34, 35].Endospores from Bacillus species were reported as early as the 19^th^ century and are known to be highly resistant to inactivation by physical parameters like wet and dry heat, UV radiation, desiccation and oxidizing agents[36]. Under favourable environmental conditions, the spores germinate into vegetative cells. For a given population of endospores, only a subset will germinate and form colonies. The actual viable endospore is likely to fall between the population which germinate and those which are viable but are not culturable [8, 37]. Counting the non culturable cells has been a challenge and hence alternative methods including real time polymerase chain reaction, fluorescent in situ hybridization,flow cytometry and propidium monoazide (PMA) real-time quantitative polymerase chain reaction have been used to enumerate culturable and VBNC bacteria of many species[14–21].

Several fluorescent dyes have been developed to stain the bacterial endospores, which were initially thought to be impermeable to intracellular dyes [38].Enumeration of Bacillus endospores atdifferent physiological conditions by FCM was described in a few studies [39, 40]. SYTO BC™ is a mixture of SYTO dyes that easily penetrates the spores and can be used to count the total number of cells in the suspension. We used CFDA as a dye to stain viable cells as B coagulans intact spores are known to have an esterase activity [41]. Propidium Iodide which can enter only dead or damaged spores was used to delineate the dead spores from live ones. Using this rationale, we were able to show that FCM can accurately enumerate the spore count in a freshly prepared suspension which was comparable to the plate count method.

VBNC can be induced by several unfavourable environmental conditions including starvation, extreme temperature and pH, oxidative stress, desiccation, and exposure to xenobiotic materials [7]. We chose acidic pH and high temperature (140°C for 5 minutes) to induce the non culturable state in *B coagulans* spores. Our earlier studies showed 2.1 log_10_ reduction in plate count at pH of 1.5 in 4 hours[32]. These extreme conditions resulted in more than a log reduction in plate count while the FCM count was not affected. Incubation of these stressed cells in nutrient medium could restore the culturability of the spores as seen by the increase in plate counts. Similar observations were reported for Bifidobacterium under bile salt stress [42].Since compression of tablets under pressure could also induce an unfavourable condition, we enumerated the spore count in tablets and capsule formulations. The spore count by plating and FCM was comparable in capsules while only minor increase in count was observed in tablets suggesting that the process of commercial formulations do not adversely affect the spore count. Addition of spores to orange juice resulted in reduction in plate count but not FCM, suggesting the existence of VBNC, further corroborating the results observed in acidic condition. These results suggest that FCM is a sensitive and powerful tool to assess the viability of *B coagulans* under stressed conditions.

Our results also show evidence of survival of B coagulans at both acidic and alkaline pH at room temperature in comparison to few other commercial probiotics which were found to lose their viability rapidly. Since the comparisons were based on FCM analysis, the count of viable bacteria included those in VBNC states as well. In comparison to non sporulating probiotics,*B*. *Coagulans* spores have an advantage on survival and shows better stability as they can also exist in the VBNC state under stressful conditions; viability being a critical factor for regulatory compliance and function of all probiotic formulations.

While VBNC state and spores are physiologically different processes, both are bacterial adaptation strategies to environmental unfavourable conditions to growth and stay viable for several years [43, 44].The extreme stability demonstrated by B coagulans spores could be because of the highly resistant endospores which have has one or more layers of proteinaceous spore coat which protect the spore from extreme conditions coupled with its ability to remain as VBNC to thrive unfavourable conditions.

*B*. *coagulans* MTCC 5856 is categorized as Generally Regarded As Safe (GRAS) for human consumption at a maximum levels of 2×10^9^ cfu/serving and 36.4×10^9^ cfu spores per day. The viable counts estimated by FCM in the commercial preparation were 20–40% higher compared to plate counts which does not exceed the maximum level for consumption per day. These results further suggest that the overages added in commercial preparations may be lowered as at present they are based on only plate counts.

In conclusion our study demonstrates for the first time that under conditions of severe environmental stress, the spores of *B*. *coagulans* MTCC 5856 remain viable but are not culturable. We also show that Flow cytometry is a fast and accurate method for evaluating viable spore count in various commercial probiotic preparations. The limitations of FCM is that the technique loses its sensitivity if the number of spores are less than ten thousand per ml and it is difficult to estimate the spores from particulate food preparations, which can clog the thin tubes used in the instrument. Further testing of different formulations by FCM will help to build concrete evidence to eventually use FCM as a preferred method to enumerate viable probiotic counts.
